# Determination of the area at risk using quantitative T2 mapping in re-perfused myocardial infarction; a comparison with late gadolinium enhancement and CINE imaging

**DOI:** 10.1186/1532-429X-15-S1-P215

**Published:** 2013-01-30

**Authors:** Tae Hoon Kim, Eui-Young Choi, Chul Hwan Park, Sung Ho Hwang, Sang Jin Kim

**Affiliations:** 1 Radiology, Gangnam Severance Hospital, Seoul, Republic of Korea; 2 Cardiology, Gangnam Severance Hospital, Seoul, Republic of Korea; 3 Radiology, Severance Hospital, Seoul, Republic of Korea

## Background

Althoug T2-weighted image (T2WI) can provide the area at risk in patients with acute myocardial infarction, the value of T2WI is still in dispute due to various limitations of T2WI. Quantitative T2 mapping can overcome the problems of T2WI and delineate myocardial edema with increased accuracy.

## Methods

Between Feb.2012, June.2012, 15 patients with acute MI underwent cardiac MRI after percutaneous primary coronary intervention (PPCI). Acute MI was diagnosed, based on EKG, coronary angiography and cardiac biomarker. In short axis view, every analyzed myocardium was subdivided into 30 sectors evenly using commercially available software (Argus; Siemens Medical Solutions, Erlangen, Germany). On CINE images, sector based myocardial contractility were calculated semi-automatically as a percentage of the systolic LV wall thickening (%, ((LV wall thicknessES - LV wall thicknessED)/LV wall thicknessED ) x 100, ES ; end-systole, ED ; end-diastole). T2 value of myocardium was measured in each sector. Infarcted zone was defined as hyper-enhanced area on LGE . On T2 mapping, AAR was defined as sectors that had T2 value (ms) > 2SD from remote myocardium. On CINE imaging, dysfunctional myocardium was defined as sectors that had LV wall thickening less than 40% or wall thickening < 80% of remote myocardium. The size (% of involving sector) of infarcted zone, AAR, and dysfunctional myocardium were calculated and then compared.

## Results

In all patients, AAR was delineated on T2 map with increased T2 values. The size of infarcted zone was smallest (24.6±11% ) and dysfunctional myocardium was largest (54.6±10.2%). The size of AAR on T2 map was between infarcted zone and dysfunctional myocardium (42.3±8%). The size differences (infarcted zone vs. AAR vs. dysfunctional myocardium) were statistically significant one another. (p<0.05).

## Conclusions

T2 map can delineate constantly myocardial edema, representing AAR in acute MI. The size of AAR quantified by T2 mapping was between infarcted zone and dysfunctional myocardium in re-perfused AMI.

